# Supplementary material for: Adaptive Laboratory Evolution of Staphylococcus aureus Resistance to Vancomycin and Daptomycin: Mutation Patterns and Cross-Resistance
Source: Antibiotics (Basel). 2023 May 18;12(5):928. doi: 10.3390/antibiotics12050928 (PMC10215302; doi:10.3390/antibiotics12050928)
Supplement: Supplementary file 1 [file antibiotics-12-00928-s001.zip › Supplemental_Figure_S2.pdf]

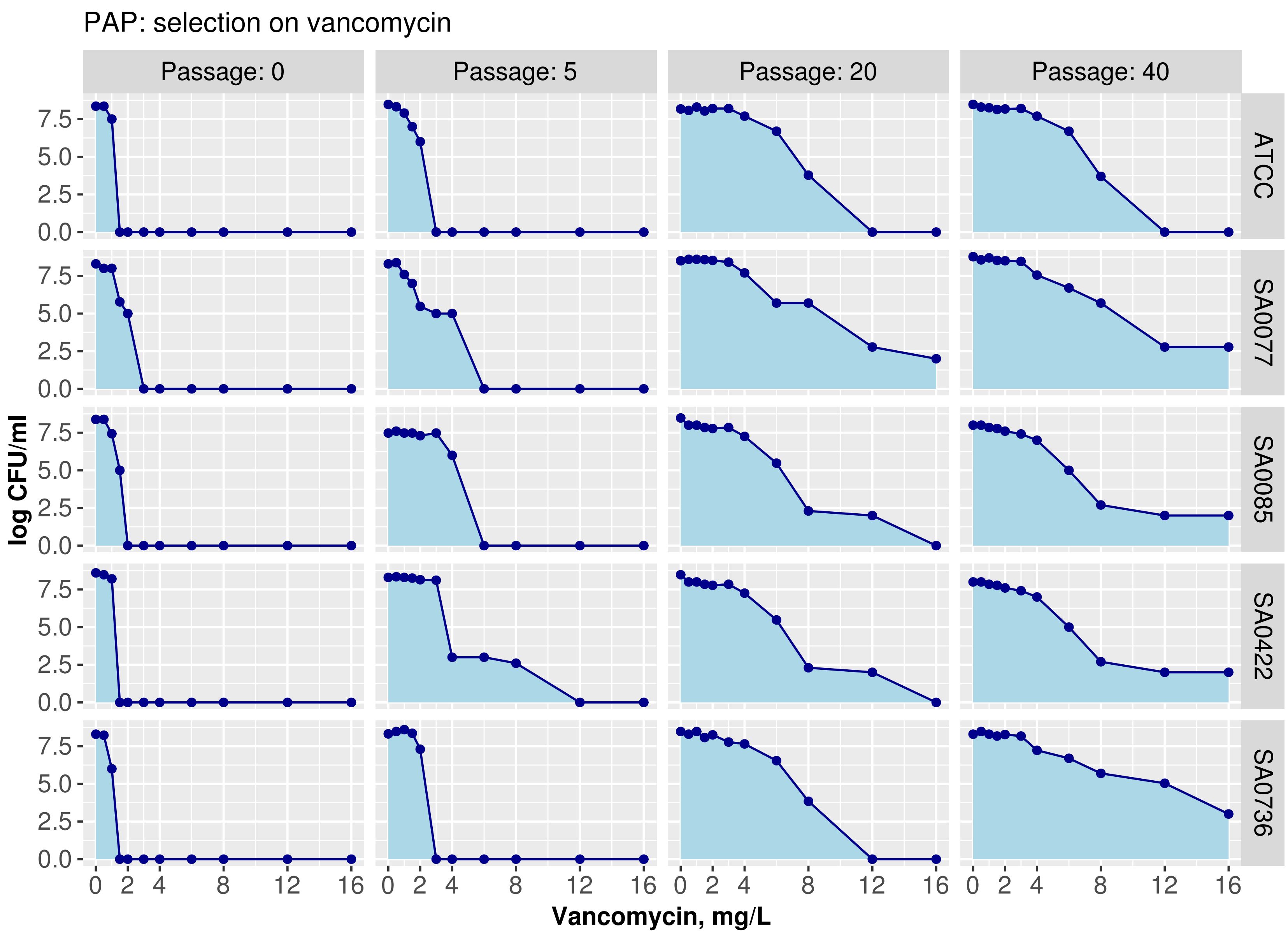

Supplemental Figure S2. Raw plots of PAP analysis with vancomycin during vancomycin resistance selection.

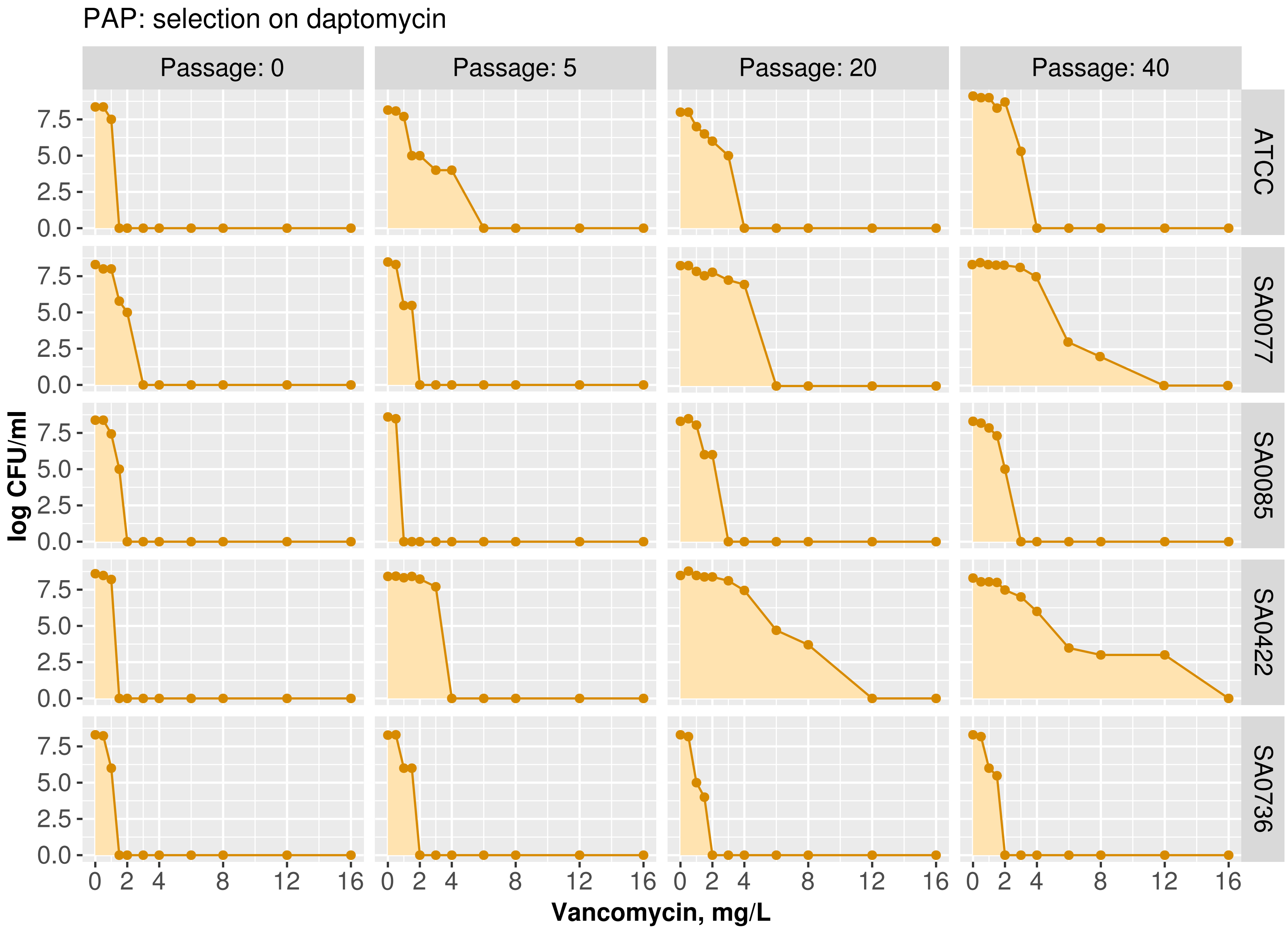

Supplemental Figure S2. Raw plots of PAP analysis with vancomycin during daptomycin resistance selection.
