# Supplementary material for: Adaptive Laboratory Evolution of Staphylococcus aureus Resistance to Vancomycin and Daptomycin: Mutation Patterns and Cross-Resistance
Source: Antibiotics (Basel). 2023 May 18;12(5):928. doi: 10.3390/antibiotics12050928 (PMC10215302; doi:10.3390/antibiotics12050928)
Supplement: Supplementary file 1 [file antibiotics-12-00928-s001.zip › Supplemental_Figure_S3.pdf]

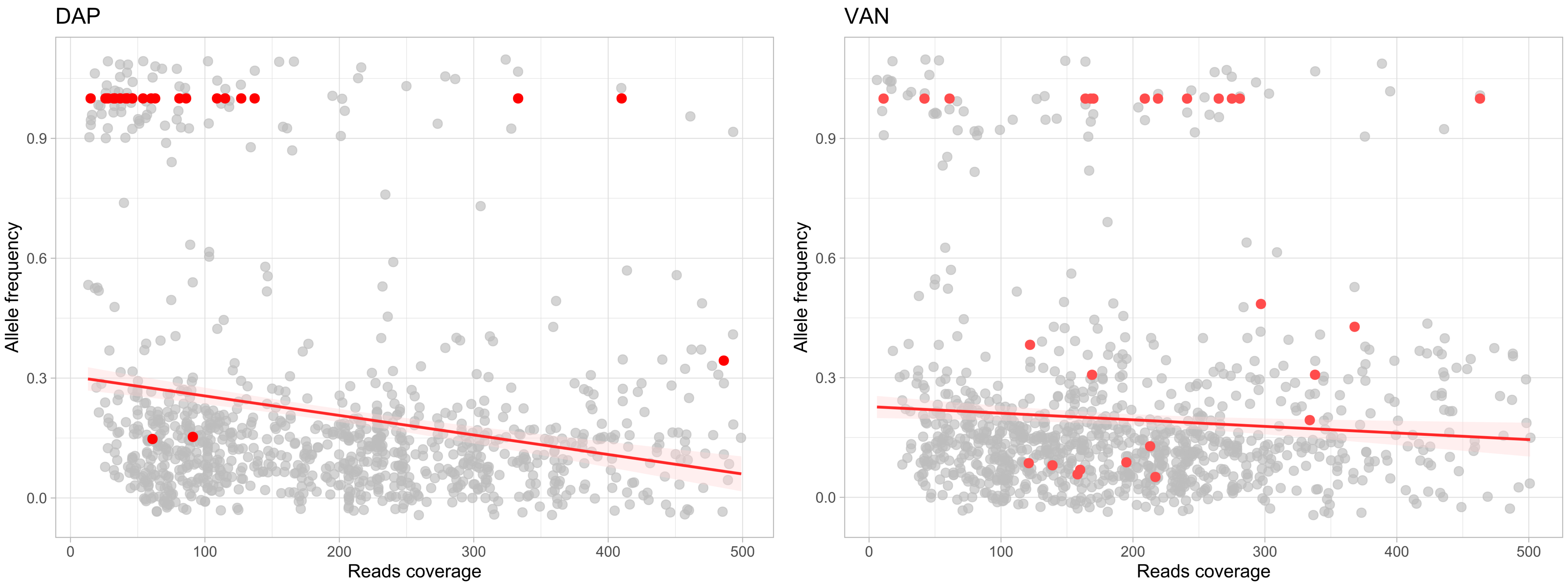

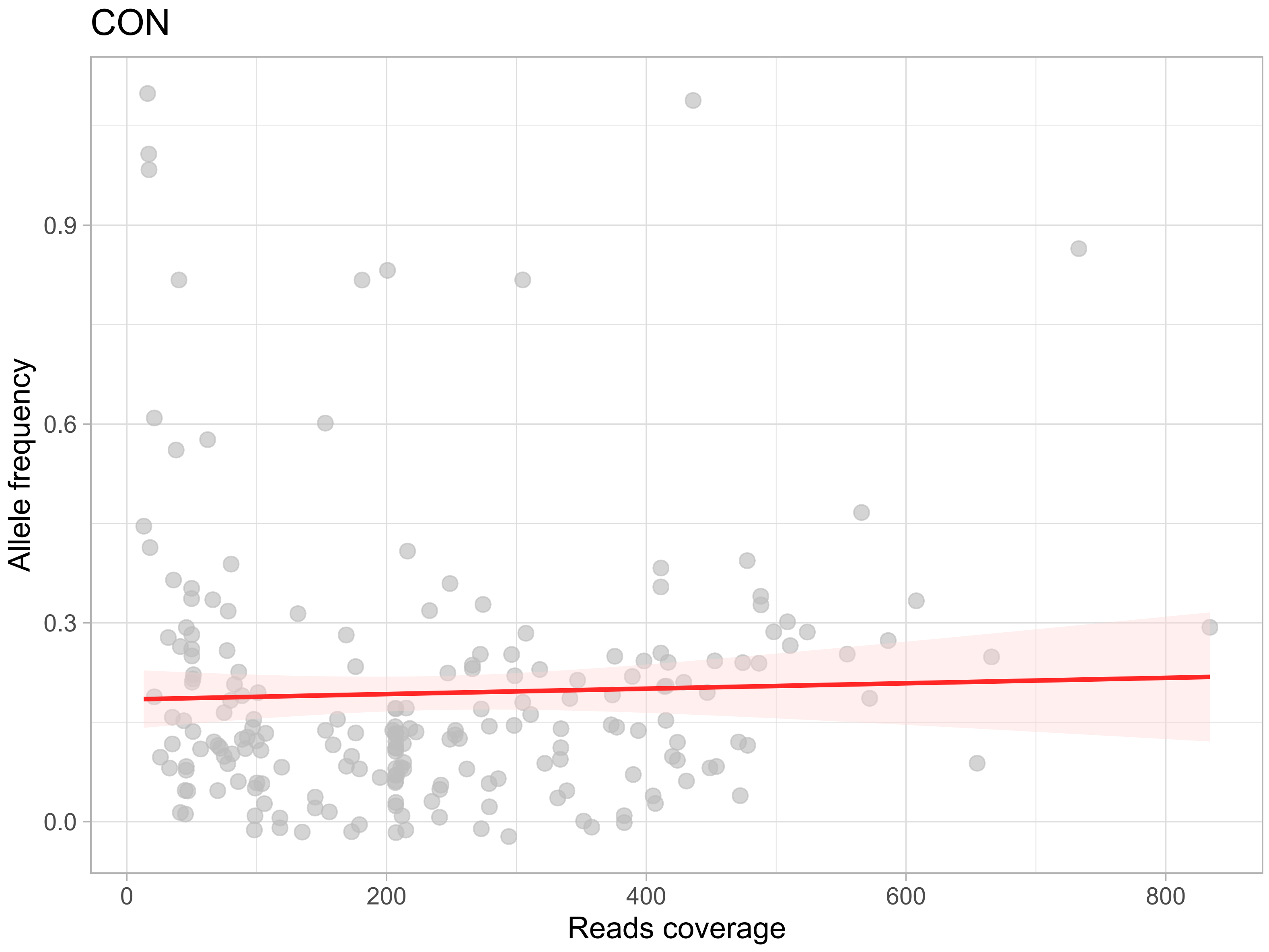

Supplemental Figure S3. Correlation analysis of read allele frequency and genome coverage. Overall sequence data from all derivative strains obtained during vancomycin (VAN), daptomycin (DAP) resistance selection, or antibiotic-free passaging (CON) from all collection time points are presented. Linear estimation (red line) was used. Red points are matched to key SNPs involved in vancomycin or daptomycin resistance.
