# Supplementary material for: Adaptive Laboratory Evolution of Staphylococcus aureus Resistance to Vancomycin and Daptomycin: Mutation Patterns and Cross-Resistance
Source: Antibiotics (Basel). 2023 May 18;12(5):928. doi: 10.3390/antibiotics12050928 (PMC10215302; doi:10.3390/antibiotics12050928)
Supplement: Supplementary file 1 [file antibiotics-12-00928-s001.zip › Supplemental_Table_S1.pdf]

Supplemental Table S1. MIC values for evolved strains and their progenitors (µg/mL).

Antibiotics	Strains																			
	ATCC 29213			SA0077				SA0085				SA0422			SA0736				RN4220	
	WT	VAN	DAP	WT	VAN	DAP	Abf	WT	VAN	DAP	Abf	WT	VAN	DAP	WT	VAN	DAP-W	DAP-Y	WT	Δ
Oxacillin	<0.5	<0.5	<0.5	512	32	256	128	256	32	256	1	512	256	2	64	64	64	16	<0.5	<0.5
Cefoxitin	4	4	2	256	128	128	64	256	4	256	4	256	256	64	64	128	64	8	4	4
Ceftaroline	.125	.125	0.25	1	1	0.5	1	1	.125	1	0.5	2	1	0.5	1	0.5	0.5	0.5	0.5	0.5
Vancomycin	0.25	8	4	1	8	4	1	1	8	4	1	1	4	2	0.5	8	4	4	0.5	0.5
Teicoplanin	0.06	8	4	0.25	8	0.25	.125	.125	8	8	.125	0.5	4	1	0.25	8	8	8	0.25	0.25
Daptomycin	.125	2	64	0.25	2	32	0.25	0.5	2	>64	0.5	0.25	2	>64	0.25	2	>64	>64	0.25	0.25
Oritivancin	0.06	4	2	<0.03	2	4	0.06	<0.03	1	1	<0.03	<0.03	0.5	0.5	<0.03	2	4	4	0.016	0.016
Dalbavancin	<0.016	2	.125	0.03	2	.125	0.03	<0.016	0.25	.125	0.03	0.03	1	0.25	0.03	1	0.5	0.5	0.03	0.03
Telavancin	<0.03	0.5	0.25	0.125	1	.125	0.03	0.06	0.25	0.25	0.06	.125	1	0.25	.125	0.5	.125	.125	0.06	0.06
Linezolid	2	2	1	1	1	1	1	4	1	4	4	2	2	1	1	2	1	1	NT	NT
Tedizolid	0.25	0.25	0.25	0.25	.125	.125	0.125	0.5	0.06	0.5	0.5	0.25	0.25	.125	.125	.125	.125	0.125	NT	NT
Tigecycline	0.03	0.06	.016	0.25	0.06	0.06	0.06	.125	.125	.125	.125	.125	.125	0.06	0.06	.125	0.06	0.06	NT	NT
Tetracycline	.125	0.5	0.06	0.06	0.06	0.06	0.06	32	.125	16	32	0.06	.125	0.06	16	16	16	16	NT	NT
Ciprofloxacin	0.25	0.25	0.25	16	64	16	32	256	32	256	256	128	64	32	128	16	64	32	NT	NT
Moxifloxacin	.125	.125	.125	2	4	2	2	8	8	8	8	2	2	2	2	4	4	2	NT	NT
Rifampicin	.004	.004	.004	.004	.004	.004	.004	>4	>4	>4	>4	.004	.004	.004	.004	.016	.004	.004	NT	NT
Gentamicin	1	1	0.25	>128	>128	>128	>128	>128	>128	>128	>128	>128	>128	>128	>128	>128	>128	>128	NT	NT
Mupirocin	1	1	1	0.5	1	0.5	1	1	1	1	1	1	1	1	1	1	0.5	1	NT	NT
Fusidic acid	0.25	0.25	.125	0.06	0.06	0.06	0.06	.125	.125	.125	.125	.125	0.06	0.06	0.06	.125	0.06	0.06	NT	NT
Erythromycin	0.25	0.5	.125	>128	>128	>128	>128	>128	>128	>128	>128	.125	0.5	.125	.125	0.5	.125	.125	NT	NT
Clindamycin	0.06	0.06	0.06	>64	>64	>64	>64	>64	>64	>64	>64	0.06	0.06	0.06	0.06	0.06	0.06	0.06	NT	NT
Trimethoprim/ sulfamethoxazole	.125	.125	0.06	0.03	0.06	0.06	.125	0.25	0.5	0.25	0.25	0.06	.125	.125	0.06	.125	.125	.125	NT	NT

Notes: WT: MICs of parental strains; VAN/DAP: MICs of derivative strains after 40 passages on vancomycin or daptomycin; Abf: passages on antibiotic-free media; RN4220—strain with deletion in SACOL1927; highlighting MICs—differences between WT and derivatives in MICs by more than one dilution; NT—not tested.
