# Supplementary material for: Adaptive Laboratory Evolution of Staphylococcus aureus Resistance to Vancomycin and Daptomycin: Mutation Patterns and Cross-Resistance
Source: Antibiotics (Basel). 2023 May 18;12(5):928. doi: 10.3390/antibiotics12050928 (PMC10215302; doi:10.3390/antibiotics12050928)
Supplement: Supplementary file 1 [file antibiotics-12-00928-s001.zip › Supplemental_Table_S2.pdf]

Supplemental Table S2. Kinetic growth parameters of parental and isogenic derivatives.

Isolate	Selection	Dt, min*		r, min^{-1}		Lag, min
		M	Q1-Q3	M	Q1-Q3	Mean
SA0077	WT	26,086	24,495 - 32,968	0,027	0,021 - 0,028	148,82
	VAN	40,073*	37,227 - 43,888	0,017*	0,016 - 0,019	168,66*
	DAP	66,124*	59,558 - 67,196	0,010*	0,010 - 0,012	228,19*
	AB free	25,034	24,321 - 31,784	0,027	0,021 - 0,028	148,82
	AB free +Ca	26,235	25,078 - 30,674	0,027	0,021 - 0,028	148,82
SA0085	WT	25,4	24,657 - 26,306	0,027	0,026 - 0,028	178,55
	VAN	42,069*	37,771 - 49,315	0,017*	0,014 - 0,018	208,3*
	DAP	39,276*	34,624 - 40,284	0,018*	0,017 - 0,020	367,01*
	AB free	24,656	23,554 - 25,987	0,027	0,026 - 0,028	178,55
	AB free +Ca	25,243	24,123 - 26,052	0,027	0,026 - 0,028	178,55
SA0422	WT	24,251	24,160 - 24,532	0,029	0,028 - 0,029	168,6
	VAN	34,157*	33,912 - 35,260	0,020*	0,020 - 0,020	178,51*
	DAP	30,858*	29,273 - 34,268	0,022*	0,020 - 0,024	198,34*
SA0736	WT	45,897	41,283 - 50,923	0,013	0,012 - 0,017	178,5
	VAN	56,807*	52,749 - 62,727	0,012	0,011 - 0,013	208,25
	DAP-W	62,020*	57,969 - 69,021	0,011	0,010 - 0,012	495,85*
	DAP-Y	63,13*	57,324 - 69,766	0,011	0,010 - 0,012	564,41*
ATCC	WT	20,629	17,417 - 23,926	0,034	0,029 - 0,040	128,92
	VAN	26,768*	26,419 - 28,411	0,026*	0,024 - 0,026	178,51
	DAP	25,615	23,403 - 32,588	0,027*	0,021 - 0,030	228,09*

Dt—doubling time, median (M), and interquartile range (Q1–Q3) are shown; r—growth rate; Lag—lag phase of kinetic growth; WT—wild-type strain prior to selection; VAN—selection with vancomycin; DAP—selection with daptomycin. For the SA0736 strain, DAP-W and DAP-Y is the white and yellow colony after the 40th passage, respectively.
