# Supplementary material for: Adaptive Laboratory Evolution of Staphylococcus aureus Resistance to Vancomycin and Daptomycin: Mutation Patterns and Cross-Resistance
Source: Antibiotics (Basel). 2023 May 18;12(5):928. doi: 10.3390/antibiotics12050928 (PMC10215302; doi:10.3390/antibiotics12050928)
Supplement: Supplementary file 1 [file antibiotics-12-00928-s001.zip › Supplemental_Table_S4.pdf]

Supplemental Table S4. Bacterial strains used in the study

Strain	Source	Origin	Genotype	Phenotype	VAN MIC	DAP MIC	Selection on
SA0077	Clinical	2011, Moscow, HA-MRSA	ST8-t008-SCCmec IVc	MRSA	1	0.25	VAN, DAP, Ab free, Ab free + Ca
SA0085	Clinical	2011, Moscow, HA-MRSA	ST239-t632-SCCmec IIIA	MRSA	1	0.5	VAN, DAP, Ab free, Ab free + Ca
SA0422	Clinical	2013, Moscow, HA-MRSA	ST8-t008-SCCmec IVc	MRSA	1	0.25	VAN, DAP
SA0736	Clinical	2014, Saint Petersburg, HA-MRSA	ST8-t008-SCCmec IVc	MRSA	0.5	0.25	VAN, DAP
ATCC 29213	Laboratory reference	Culti-Loops™, Thermo Scientific™	ST5-t002	MSSA	0.25	0.125	VAN, DAP
RN4220	Laboratory reference			MSSA	0.5	0.25	Used for genome editing

VAN – selection on vancomycin, DAP – selection on daptomycin, Ab free – selection on antibiotic free medium, Ab free + Ca – selection on antibiotic free medium supplemented 50 mg/L of calcium.
