# Supplementary material for: Adaptive Laboratory Evolution of Staphylococcus aureus Resistance to Vancomycin and Daptomycin: Mutation Patterns and Cross-Resistance
Source: Antibiotics (Basel). 2023 May 18;12(5):928. doi: 10.3390/antibiotics12050928 (PMC10215302; doi:10.3390/antibiotics12050928)
Supplement: Supplementary file 1 [file antibiotics-12-00928-s001.zip › Supplemental_Table_S5.pdf]

Supplemental Table S5. Oligos used for genome editing of *S. aureus* RN4220

Oligonucleotide name	Oligonucleotide sequence	Notes
Gibson_for_ SACOL1927	PO_4-agctcCCAGCAATGGCTTCTACGTTG	targets PAM internal to SACOL1927 deletion
Gibson_rev_ SACOL1927	PO_4-aaaacAACGTAGAAGCCATTGCTGGG	targets PAM internal to SACOL1927 deletion
90bp_sav_del	A*T*A*T*TATGAAATGAGTGAACTGATATG GACACTGCAACACATATCTTCGCATCATAGA GGTATCACGCATTCCATCCCTTTCACAATACT	90bp probe with 4 5' phosphothiorate bonds to introduce 146 bp deletion in SACOL1927 gene, asterisks - phosphothiorate bonds
sav_for	GGATTGGTCGTTTGTCGC	Oligo for PCR SACOL1927 gene fragment
sav_rev	TGAAATGGGTTGGTTCCAC	Oligo for PCR SACOL1927 gene fragment
